# Medical Societies

**Published:** 1867-08

**Authors:** 


### *Medical Societies.*

Thanks are due to the Secretaries who have forwarded Transactions, but the present pressure upon our pages renders it necessary to defer further notice in this number.

